# Exercise Training Prevents Decrease in Luminal Capillary Diameter of Skeletal Muscles in Rats with Type 2 Diabetes

**DOI:** 10.1100/2012/645891

**Published:** 2012-08-13

**Authors:** Takeshi Morifuji, Shinichiro Murakami, Naoto Fujita, Hiroyo Kondo, Hidemi Fujino

**Affiliations:** ^1^Department of Rehabilitation Science, Kobe University Graduate School of Health Sciences, 7-10-2 Tomogaoka, Suma-ku, Kobe 654-0142, Japan; ^2^Department of Physical Therapy, Kansai Medical College, 3-27 Suehirocho, Kita-ku, Osaka 530-0053, Japan; ^3^Department of Physical Therapy, Himeji Dokkyo University, 7-2-1 Kamiohno, Himeji 670-8524, Japan; ^4^Department of Food Sciences and Nutrition, Nagoya Women's University, 3-40 Sioji-cho, Mizuho-ku, Nagoya 467-8610, Japan

## Abstract

The purpose of this study was to examine whether exercise training can prevent microangiopathy of skeletal muscles in rats with type 2 diabetes and if succinate dehydrogenase (SDH) activity, an indicator of mitochondrial oxidative enzyme activity, is involved in the prevention of microangiopathy. Six-week-old male Goto-Kakizaki (GK) rats and age-matched male Wistar rats (control group (Con)) were used. GK rats were randomly assigned to nonexercise (DB) and exercise (DBEx) groups. The DBEx group was trained on a treadmill 5 times a week for 3 weeks. No significant differences in the capillary-to-fibre ratio or the capillary density were observed between the 3 groups. The luminal capillary diameter of the DB group was significantly lower than that of the Con group, whereas the capillary diameter of the DBEx group was significantly higher than that of the DB group. In addition, SDH activity was significantly higher in the DBEx group than in the Con and DB groups. Microangiopathy of skeletal muscles in type 2 diabetes was correlated with a decrease in the luminal capillary diameter, which was prevented by exercise training. Thus, the mitochondrial oxidative capacity appears to be involved in the overall mechanism by which exercise prevents microangiopathy.

## 1. Introduction

Type 2 diabetes is a significant public health problem worldwide [1]. Diabetes is an important risk factor for macro- and microangiopathy vascular disorders, which contribute to the development of severe complications that may lead to major disability [2, 3]. A number of previous studies have demonstrated that hyperglycaemia induces structural remodelling of capillaries in the muscles of diabetic humans and rodents [4–6]. Skeletal muscle capillaries have frequently been evaluated histologically to determine the number of capillaries per individual muscle fibre, that is, the capillary-to-fibre (C/F) ratio, and the number of capillaries per unit area, that is, the capillary density [7, 8]. Previous studies have shown that both the C/F ratio [5] and the capillary density [4, 6] of skeletal muscles are reduced in diabetic humans and rodents. In addition, the capillary diameter of skeletal muscles is decreased in rats with streptozotocin-induced diabetes [5, 9].

In general, exercise training is recommended for diabetes, and previous studies have shown that exercise is effective for glycaemic control [10] and insulin sensitivity in skeletal muscles [11]. However, the effect of exercise training on microangiopathy of skeletal muscles is still unclear and the opinions regarding this effect are conflicting. Although it has been reported that exercise training did not prevent microangiopathy in both type 1 diabetic humans [12] and rodents [13], exercise training was shown to prevent microangiopathy in type 2 diabetic rodents [14]. In addition, the effect of exercise training on the capillary diameter of skeletal muscles in diabetic humans and rodents is poorly understood because most studies have evaluated only the number of capillaries per unit area of tissue, that is, the C/F ratio and capillary density [12–14]. Thus, the effect of exercise training on microangiopathy of skeletal muscles in diabetics is not well understood. Exercise training usually increases oxidative enzyme activity and capillary supply in skeletal muscles [8]. In addition, endurance training leads to an increase in oxidative enzyme activity prior to the increase in capillary supply [15]. We hypothesised that exercise training increases the levels of succinate dehydrogenase (SDH) activity, an indicator of mitochondrial oxidative enzyme activity [16, 17], which leads to an increase in capillary supply and prevents microangiopathy of skeletal muscles in diabetics. Therefore, the purpose of this study was to histologically examine (1) whether exercise training prevents microangiopathy of skeletal muscles in rats with type 2 diabetes and (2) if SDH activity is involved in the prevention of capillary regression.

## 2. Materials and Methods

### 2.1. Animals and Exercise Program

Six-week-old male Goto-Kakizaki (GK; *n* = 14) and age-matched male Wistar rats (*n* = 7) (control group (Con)) were used in the present study. The GK rat is a nonobese, hyperglycaemic, insulin-resistant rat strain that was developed by selective breeding of an outbred colony of Wistar rats with high glucose levels [18]. The GK rats were randomly assigned to nonexercise (DB) (*n* = 7) or exercise (DBEx) (*n* = 7) groups.

Animals in the DBEx group ran continuously for 1 h per day on a treadmill at 15 m/min (0% gradient) 5 times a week for 3 weeks. The animals in the DBEx group were familiarised with the treadmill for 5–10 min per day for 1 week before starting the exercise program. The blood lactate levels in the DBEx group were measured from the samples collected from the tail vein by using a blood lactate test meter (Lactate Pro; Arkray, Shiga, Japan) before and after exercise. This exercise was considered aerobic in terms of intensity because the levels of blood lactate did not change significantly (<2 mmol/L) before and after exercise. Animals were housed in a temperature-controlled room at 22 ± 2°C with a light-dark cycle of 12 h and were maintained on standard rat chow and water *ad libitum*. All experiments were conducted in accordance with the National Institutes of Health (NIH) Guide for the Care and Use of Laboratory Animals (National Research Council, 1996) and were approved by the Animal Care and Use Committee of Kobe University.

### 2.2. Muscle Sample Preparation

At 9 weeks of age, animals were anaesthetised with pentobarbital sodium (50 mg/kg; i.p.), and the left soleus muscles were excised. Muscle samples were immediately frozen in isopentane (precooled in liquid nitrogen) and stored at −80°C. The luminal diameter of the muscle capillaries was visualised by confocal laser scanning microscopy, as previously described [19]. Briefly, the abdomen was opened, a perfusion apparatus catheter was inserted into the abdominal aorta to maintain the root in the right hind limb, and the left common iliac artery was ligated. Thereafter, 0.1 M phosphate buffer (pH 7.2) containing 10,000 IU/L of heparin was perfused for 3 min in order to wash out intravascular blood and to maximally induce vasodilation with a perfusion pressure of 110–120 mm Hg through the catheter. Contrast medium (42°C) consisting of 1% fluorescent material (PUSR80; Mitsubishi Pencil, Tokyo, Japan), 8% gelatine (Nacalai Tesque, Kyoto, Japan), and distilled water was subsequently administered into the circulation of the right soleus muscle. After perfusion with the contrast medium, the entire body of the animal was quickly immersed into cold saline for 10 min, which enabled us to completely fill the entire microvasculature under a physiologically relevant perfusion pressure [20]. Finally, the right soleus muscles perfused with contrast medium were excised, frozen in isopentane (precooled in liquid nitrogen), and stored at −80°C until further analysis.

### 2.3. Glucose Measurement

Glucose levels were determined using a blood glucose monitoring system (Precision Xceed, Abbot Laboratories, USA) on blood samples obtained from the abdominal veins of 9-week-old rats.

### 2.4. Histological Analyses

The left soleus muscle was sliced into 10 *μ*m thick transverse sections with a cryostat microtome (CM3050; Leica Microsystems, Mannheim, Germany) at −20°C, and the sections were dried at room temperature for 30 min. Several sections were stained to determine the levels of alkaline phosphatase (AP) and to visualise the capillaries in the skeletal muscle. For AP staining, the sections were incubated for 60 min at 37°C in 0.1% 5-bromo-4-chloro-3-indolyl phosphate *p*-toluidine salt and 0.1% nitro blue tetrazolium in 0.2 M borate buffer and fixed with 4% paraformaldehyde [21]. The sections were examined with a light microscope (BX51; Olympus, Tokyo, Japan), and images were obtained using a charge coupled device (CCD) camera (VB-7000; Keyence, Osaka, Japan). The C/F ratio and capillary density were measured for 2 microscopic images selected at random from AP-stained sections, and all measurements were subsequently calculated using the ImageJ software program. The C/F ratio was determined by counting all capillaries and fibres in a microscopic image, and the capillary density was determined by counting all capillaries in a 1 mm^2^ cross-sectional area.

SDH activity was measured for the stained sections. SDH staining was performed by incubating the sections for 30 min at 37°C in 0.05% nitro blue tetrazolium and 0.05 M sodium succinate in 0.05 M phosphate buffer (pH 7.5) [21]. Cross-sectional tissue images were examined with a light microscope and images were obtained using a CCD camera. Two microscopic images were selected randomly from each section, and all muscle fibres in each image were analysed to determine the SDH activity in the soleus muscle. SDH activity was calculated as the mean optical density (OD) by using the ImageJ software program. All values represent fold changes relative to the Con group.

The right soleus muscle was sliced into 5 *μ*m thick transverse sections with a cryostat microtome at −20°C, and sections were used to determine the capillary luminal diameter in the soleus muscle. The contrast medium-perfused sections were scanned and visualised using the fluorescent mode of a confocal laser-scanning microscope (CLSM) (TCS-SP; Leica Instruments, Manheim, Germany) with an argon laser (488 nm). Subsequently, 100 capillaries per muscle were measured to determine the luminal diameter by using the ImageJ software program.

### 2.5. Statistical Analyses

All data are expressed as the mean ± standard error. Overall differences were determined using one-way analysis of variance (ANOVA). If ANOVA revealed significance, group differences were determined using the Tukey post hoc test. *P* values <0.05 were considered statistically significant.

## 3. Results

### 3.1. Blood Glucose Levels

The mean blood glucose levels were 117.6 ± 7.1 mg/dL in the Con group, 256.7 ± 15.1 mg/dL in the DB group, and 281.1 ± 22.7 mg/dL in the DBEx group (Table 1). These values were significantly higher in the DB and DBEx groups than in the Con group, but no significant difference was found between the DB and DBEx groups.

### 3.2. C/F Ratio and Capillary Density

**Figure 1:**
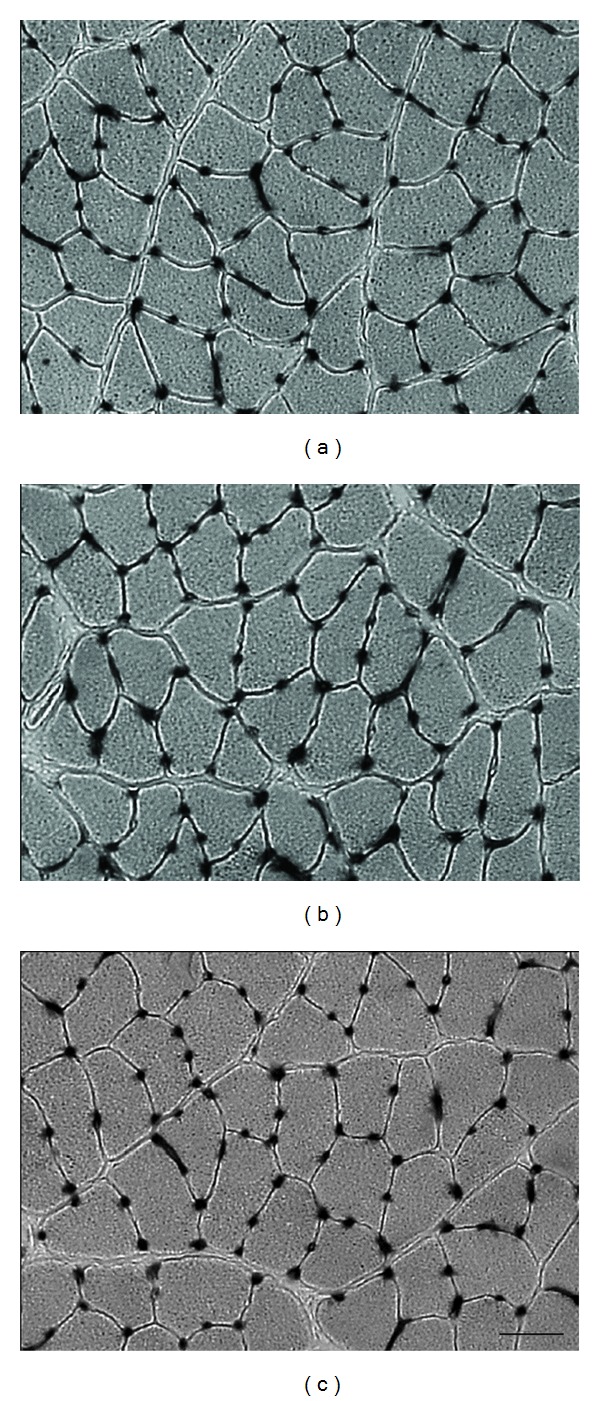
Alkaline phosphatase staining of the soleus muscle. (a) Control group; (b) diabetes group; (c) diabetes with exercise group. The capillaries are arranged around the muscle fibres and are visible as dark spots. Bar = 50 *μ*m.

The capillaries (visualised as dark spots) were similarly arranged around the muscle fibres in all 3 groups (Figure 1). The mean C/F ratio values were 2.1 ± 0.1 in the Con group, 2.3 ± 0.1 in the DB group, and 2.2 ± 0.1 in the DBEx group, with no significant difference between the 3 groups (Table 1). The mean capillary density values were 975.9 ± 39.1/mm^2^ in the Con group, 976.3 ± 42.2/mm^2^ in the DB group, and 888 ± 18.1/mm^2^ in the DBEx group; these values were not statistically different between the 3 groups (Table 1).

### 3.3. Capillary Luminal Diameter

**Figure 2:**
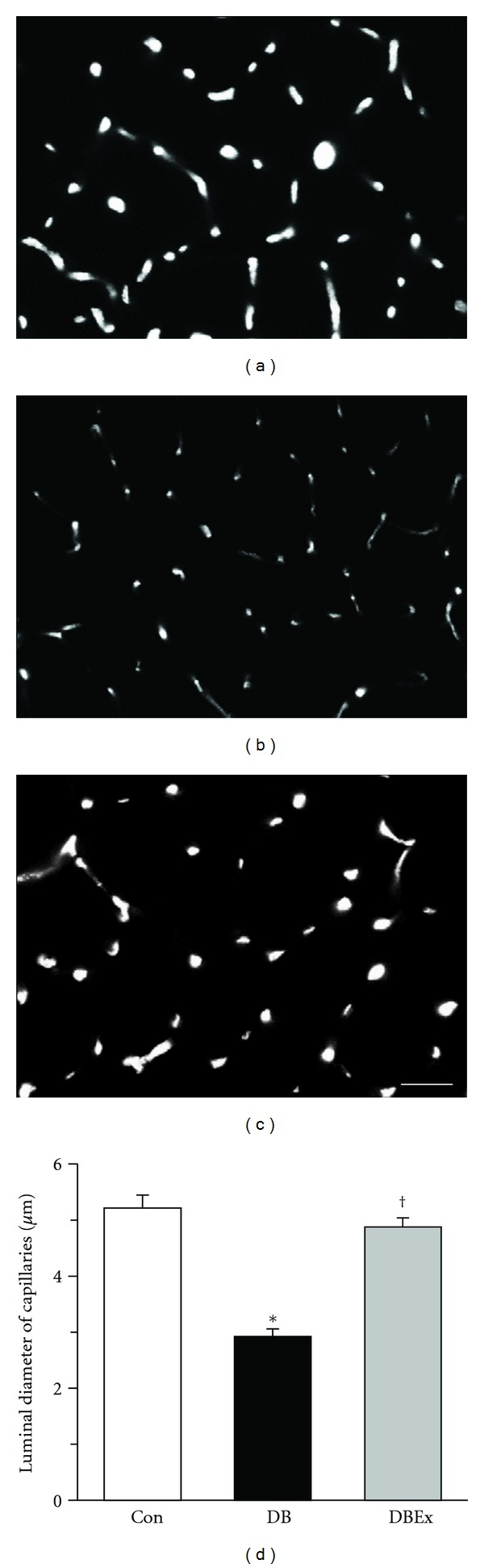
(a)–(c) Confocal scanning-laser microscope of transverse sections of the soleus muscle from the control (Con, (a)), diabetes (DB, (b)), and diabetes with exercise (DBEx, (c)) groups. The capillaries are visible as light spots. (d) The luminal diameter of capillaries in the Con, DB, and DBEx groups. Values are the mean ± standard error. ∗ and † indicate significant differences in the Con and DB groups, respectively, at *P* < 0.05. Bar = 50 *μ*m.

**Figure 3:**
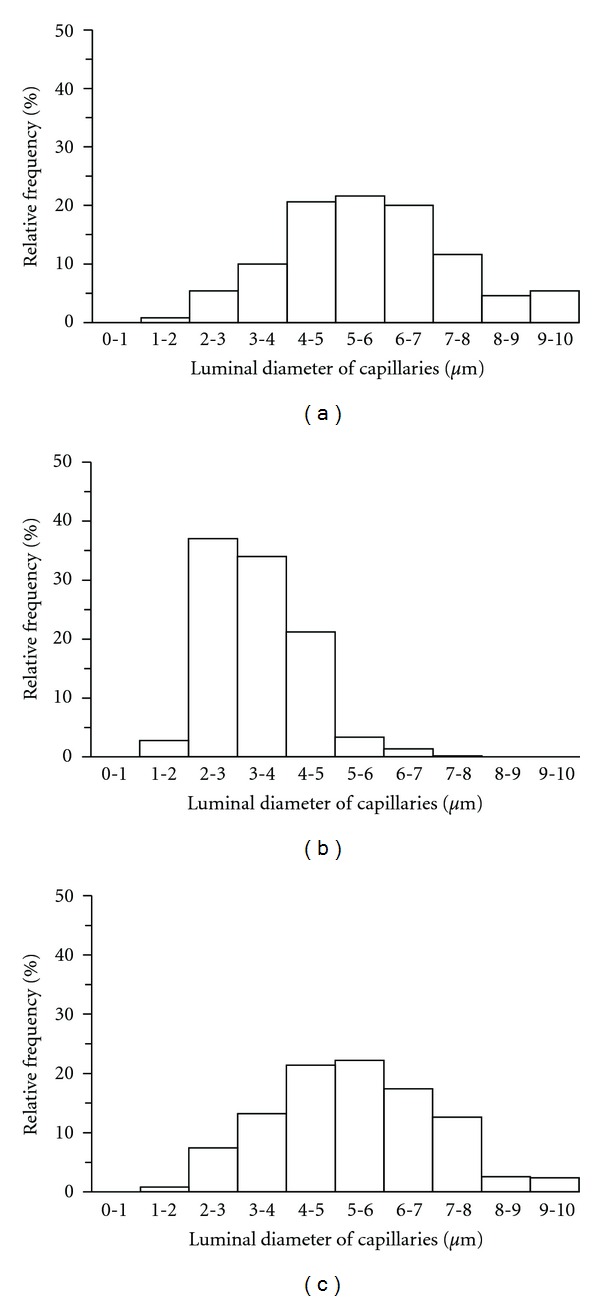
Frequency distributions of capillary luminal diameters in the soleus muscle. (a) Control group; (b) diabetes group; (c) diabetes with exercise group.

The visualization of the luminal diameters of the capillaries as light spots using CLSM revealed that the capillary diameter was larger in the Con and DBEx groups than in the DB group (Figures 2(a)–2(c)). The mean capillary luminal diameter values were 5.2 ± 0.2 *μ*m, 2.9 ± 0.2 *μ*m, and 4.9 ± 0.2 *μ*m for the Con, DB, and DBEx groups, respectively. The value for the DB group was significantly lower than the values of the Con and DBEx groups (Figure 2(d)), but no statistically significant difference was observed between the Con and DBEx groups. The frequency distribution of the capillary luminal diameters for each group is shown as a histogram in Figure 3. The histogram for the DB group was shifted to the left as compared to the Con group, which indicates an increased distribution of capillaries with reduced diameter. However, the histogram for the DBEx group was similar to that of the Con group. It was previously reported that the critical diameter at which erythrocytes cannot flow through capillaries is 2.5 *μ*m [22]. We found that the rate of capillaries with a luminal diameter of <2.5 *μ*m was 6%, 40%, and 8% for the Con, DB, and DBEx groups, respectively.

### 3.4. SDH Activity

**Figure 4:**
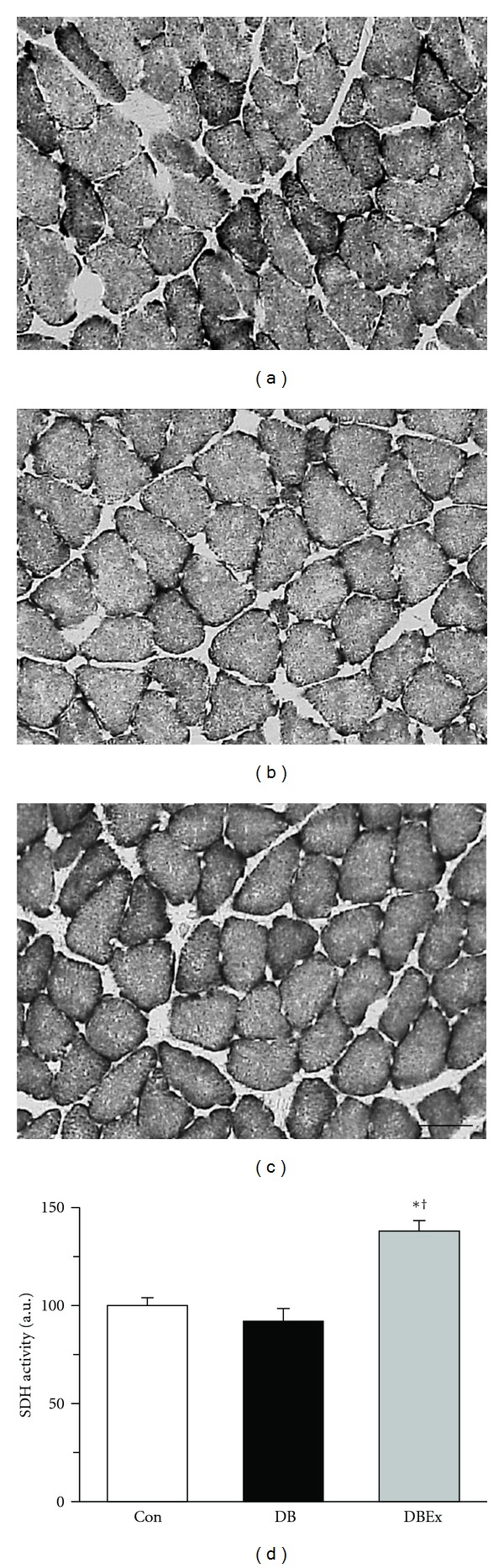
(a)–(c) Succinate dehydrogenase (SDH) staining of transverse sections of the soleus muscle from the control (Con, (a)), diabetes (DB, (b)), and diabetes with exercise (DBEx, (c)) groups. Staining intensity is directly related to SDH activity; darker muscle fibres indicate higher activity. (d) SDH activity in the Con, DB, and DBEx groups. au: arbitrary unit. All values indicate fold changes relative to the Con group. Bars represent standard error. ∗ and † indicate significant differences in the Con and DB groups, respectively, at *P* < 0.05. Bar = 50 *μ*m.

The muscle fibres were darker in the DBEx group than in both the Con and DB groups (Figures 4(a)–4(c)). The mean SDH activity values were 91.9 ± 6.5% and 138.0 ± 5.3% of the Con group (100 ± 4.1%) for the DB and DBEx groups, respectively. Moreover, the SDH activity value was significantly higher in the DBEx group than in the Con and DB groups (Figure 4(d)).

## 4. Discussion

The present study demonstrated that exercise training has a preventive effect on microangiopathy of skeletal muscles in rats with type 2 diabetes. Although the capillary luminal diameter of skeletal muscles was decreased in rats with type 2 diabetes, exercise training restored the capillary luminal diameter. Thus, exercise training was useful for preventing diabetes-induced degeneration of skeletal muscle capillaries.

The capillary luminal diameter was significantly smaller in the DB group than in the Con group, which is consistent with previous studies using animal models [5, 9]. However, neither the C/F ratio nor the capillary density in the DB group demonstrated a significant decrease compared with the Con group, which differs from both the study that used animal models [5] and studies that used human models [4, 6]. These inconsistent results may have resulted from differences in the duration and extent of exposure to hyperglycaemia. GK rats show slightly elevated glucose levels at 5 weeks of age [23]. The GK rats used in the present study were exposed to mild hyperglycaemia for 4 weeks only,and their glucose level was 256.7 ± 15.1 mg/dL. In contrast, the previous animal study [5] used diabetic rats 6–8 weeks after injection of streptozotocin to induce diabetes. Thus, these rats were exposed to severe hyperglycaemia for a longer period than the animals in our study, and the corresponding glucose level was 410 ± 28 mg/dL. Similarly, in previous studies in human models [4, 6], it was hypothesised that diabetic humans are exposed to long-term hyperglycaemia. We speculate that the decrease in capillary luminal diameter occurs earlier than the decrease in the number of capillaries. In particular, the decrease in capillary luminal diameter presumably occurs during the early stages of microangiopathy in type 2 diabetes.

A decrease in the capillary luminal diameter leads to a corresponding decrease in the oxygen supply to muscle fibres. The mean diameter of capillaries in the soleus muscle of 34-week-old male Wistar rats was reportedly 5.3 *μ*m [7], which is in agreement with the findings from the Con group in the present study (5.2 *μ*m). However, the mean capillary luminal diameter value decreased to 2.9 *μ*m in the DB group. Assuming that the previous finding that erythrocytes cannot flow through capillaries <2.5 *μ*m in diameter [22] is valid, the percentage of capillaries too small to permit the passage of erythrocytes in our study was 40% in the DB group compared with 6% in the Con group. In the DB group, the obstruction of erythrocytes in skeletal muscle capillaries would cause a considerable decrease in the oxygen supply to the muscle fibres. Thus, our findings suggest that a decrease in the capillary luminal diameter of skeletal muscles leads to a decline in exercise tolerance and serious complications in type 2 diabetes.

Based on the findings of previous studies, we can speculate on the mechanism of microangiopathy in the present study. Hyperglycaemia induces the production of advanced glycosylation end products (AGEs), which accumulate in the vasculature and are associated with vascular complications [24, 25]. In rodents with type 1 diabetes, microangiopathy of skeletal muscles is accompanied by reduced expression of VEGF [13, 26]. Therefore, we hypothesise that changes in the level of AGEs and/or VEGF caused the observed decrease in the capillary luminal diameter of the soleus muscle in the DB group. Further studies on this subject are required to confirm the mechanism of microangiopathy in type 2 diabetes.

The mean capillary luminal diameter value was 4.9 *μ*m in the DBEx exercise group compared with 2.9 *μ*m in the DB group. This result supports a previous study [14] showing that exercise training prevented microangiopathy of skeletal muscles. According to the findings of a previous study [22], erythrocytes would not pass through 8% of capillaries in the DBEx group compared to 40% in the DB group. This indicates that the oxygen supply to the muscle fibres was maintained in the DBEx group, which is supported by our finding that the mean of SDH activity value was significantly higher in the DBEx group than in the Con and DB groups. A previous study reported that the capillary supply in skeletal muscle adapts to meet the demands of oxidative metabolism [15]. In the DB group, the capillary luminal diameter was reduced because of microangiopathy. Since the rats in the DB group were sedentary, their muscle fibres did not require much oxygen and the SDH activity remained low. In contrast, the DBEx group required a high amount of oxygen because of the exercise training, which resulted in increased SDH activity. Accordingly, the capillary luminal diameter in the DBEx group was maintained to meet the increased oxygen demands of the muscle fibres.

We showed that exercise training could prevent microangiopathy in type 2 diabetes and that the increase in SDH activity participated in this effect. It was previously shown that exercise training in diabetic rodents results in a decrease in plasma AGEs without a decline in plasma glucose [27] and an increase in the concentration of VEGF in skeletal muscles [28]. We speculate that increased VEGF expression and decreased production of AGEs in vascular endothelial cells induced by exercise training prevents microangiopathy of skeletal muscles in rats with type 2 diabetes, without a decrease in glucose levels.

In the present study, the mean blood glucose value was not significantly different between the DB and DBEx groups, indicating that exercise training did not prevent the blood glucose level from rising in type 2 diabetic rodents. In general, dietary restriction is recommended in addition to exercise in order to control the glucose level in type 2 diabetes. However, dietary restriction was not addressed in the present study. A previous study reported that physical training without dietary restriction in type 2 diabetic patients did not reduce blood glucose levels [29]. Similarly, the endurance exercise performed by rodents in the DBEx group in the present study did not result in a reduction in blood glucose levels. Thus, we suggest that exercise training cannot reduce blood glucose levels in the absence of dietary restrictions.

On examining the effect of exercise training on microangiopathy of skeletal muscle in type 2 diabetes at a histological level, we found that early microangiopathy of skeletal muscles was correlated with a decrease in the diameter, but not with the number, of capillaries. To our knowledge, this is the first study to show that exercise training prevents a decrease in the capillary diameter of skeletal muscles according to the progression of type 2 diabetes. In addition, it is likely that the mitochondrial oxidative capacity is related to the mechanism by which exercise prevents capillary regression. These results suggest that exercise training prevents serious complications caused by diabetic microangiopathy, despite high blood glucose levels. However, because the present study was conducted in experimental animals and the results cannot be generalised to humans, validation of our findings in humans will be necessary in future. Further investigation of the proteins involved in the mitochondrial oxidative capacity is also required to support the results of the present study.

## Figures and Tables

**Table 1:** Blood glucose level, capillary-to-fibre ratio, and capillary density in the soleus muscle.

	Con	DB	DBEx
Blood glucose level (mg/dL)	117.6 ± 7.1	256.7 ± 15.1^∗^	281.1 ± 22.7^∗^
C/F ratio	2.1 ± 0.1	2.3 ± 0.1	2.2 ± 0.1
CD (no/mm^2^)	975.9 ± 39.1	976.3 ± 42.2	888 ± 18.1

Values are the mean ± standard error. Con: control group; DB: diabetes group; DBEx: diabetes with exercise group. C/F ratio: the capillary-to-fibre ratio; CD: capillary density. ^∗^Represents significant difference in the Con group, at *P* < 0.05.
